# An inconvenient discussion

**DOI:** 10.1111/medu.13689

**Published:** 2018-10-21

**Authors:** Pim W Teunissen

**Affiliations:** ^1^ School of Health Professions Education Maastricht University The Netherlands

## Abstract

This commentary argues for the importance of informing patients about their participation in medical education when being cared for by trainees.

Life is full of choices, from deciding what to wear when you get up in the morning to choosing what to eat for dinner in the evening. For health care professionals, work also means making choices, some simple but also some very hard ones. Deciding if a diagnostic test needs to be ordered, and if so which one, what medication to start a patient on and in what dosage, or deciding who should be invited to a multidisciplinary meeting about a complex case, are all in a day's work. Deciding what to discuss with patients regarding their contribution to medical education is an issue that deserves more attention.

In this issue of *Medical Education,* Porteous and colleagues describe a research approach to help understand how health care professionals (in training) make choices.^1^ They describe ‘discrete choice experiments’ as an approach with roots in economics that can be helpful to understand the value health professionals and trainees place on the different attributes connected to the choices they face. Next to some illustrative examples of how discrete choice experiments could be applied to issues related to health professionals’ education, they provide one example of how attributes of different health services, such as ‘distance to clinic, waiting time, consultation time and healthcare professional seen’, might impact patients’ preferences for one service over another. In this commentary, I solely focus on patients, their preferences and choices. I draw attention to our field's need to understand more about patients’ values and choices in relation to being looked after by medical students and residents.

In many countries, trainees are the backbone of the health care system.^2^ When a patient goes to a teaching hospital, that is a hospital where medical students and residents are part of the workforce, it is very likely that learners at various levels of training contribute to the collective effort that results in that individual's care. This ranges from taking a history by a junior medical student to a resident performing a complex procedure. Apart from avoiding trainees by going to a (non‐teaching) community hospital or a private hospital, patients in teaching hospitals should get the opportunity to decide if they want to contribute to medical education or not. For instance, the American Medical Association (AMA) states in their Code of Medical Ethics Opinion 9.2.1 that ‘all physicians share an obligation to ensure that patients are aware that medical students may participate in their care and have the opportunity to decline care from students’.^3^ Patients should be informed ‘about the identity and training status of individuals involved in care’.^3^

Patients should get the opportunity to decide if they want to contribute to medical education

We know that being informed matters to patients, especially when it may entail trainees performing or practising sensitive procedures, such as a rectal or pelvic examination. Patients find being informed even more important when learners are involved while they are temporarily incapacitated, such as during anaesthesia for a surgical procedure.^4^ A systematic review published in 2015 that looked at patients’ attitudes towards medical student participation across specialties found 59 relevant, but overall low‐quality, studies that used 61(!) evaluation instruments to determine patients’ attitudes.^5^ The variation of acceptance of active medical student involvement varied from 7% (allow a medical student to perform a lumbar puncture for the first time) to 100% (allow a medical student to put on a splint) depending on the sensitivity and risk of the task and presence of direct supervision. Patients’ satisfaction with their care did not appear to be influenced by medical student participation. A subset of papers also explored patients’ reasons for willingness or refusal to contribute to medical education. The three most common reasons for accepting medical student participation were a desire to contribute to education, a perceived higher quality of care and a perceived increase in the knowledge obtained during the visit. The top three reasons for refusal were a desire for privacy, increased length of the office visit and gender of the medical student, with a preference for female students in specialties such as obstetrics and gynaecology.^5^

Access to opportunities for active participation is essential for medical students’ and residents’ learning and professional development.^6^ Recent work reminded us that active involvement in patient care happens in only a small proportion of all possible learning opportunities that practice offers.^7^ The first chance to keep the pool of learning opportunities as large as possible is to get patients’ consent on active trainee involvement. Yet, it remains unclear how to best inform patients of medical student and resident participation.^5^ Who should inform patients? There are indications that, compared with nurses, when attending physicians inform patients, this may lead to much lower willingness to accept medical students’ involvement.^8^ What needs to be discussed with patients regarding trainees’ participation? The AMA suggests that the first thing to do is to ‘convey to the patient the benefits of having medical students participate in their care’.^3^ A recently published observational study in the USA on 30‐day mortality rates did indeed indicate that patients

It remains unclear how to best inform patients of medical student and resident participation

might be better off in major teaching hospitals versus non‐teaching hospitals.^9^ But what do we tell individual patients? What are the potential benefits for an individual patient in a specific hospital if a specific task or procedure is performed by a specific trainee? Perhaps, emphasising an individual patient's benefits is the wrong path. After all, most patients agree not because they think they themselves will benefit but because they want to contribute to a greater good: education of the future health care workforce.^5^ To answer these questions, a programme of research is necessary. When there is more insight into the type of information that patients find relevant when considering whether to consent to contribute to medical education, a next step could be to use discrete choice experiments to understand how these different attributes are weighed and valued relative to each other.^1^

Having a conversation with patients about their contribution to medical education starts with disclosing the presence of learners. Unfortunately, research indicates that our students might, through exposure to current practice, become less inclined to seek informed consent.^10^ This is a serious ethical issue, as Devettere notes in his book on health care ethics.^11^ First of all

Our students might, through exposure to current practice, become less inclined to seek informed consent

because not disclosing this information ‘disenfranchises patients’ and second because it undermines the trust the public has in the medical profession.^11^ Trust is essential. At a time when the margins within which clinical educators need to balance patient safety with allowing trainees to learn from real practice seem to be getting smaller, a further erosion of trust could lead to a breakdown of training in actual practice.

A further erosion of trust could lead to a breakdown of training in actual practice

The field of health professions education should be at the forefront of a societal discussion on this topic. Large educational changes, such as the adoption of outcome‐based education, programmatic assessment and the use of entrustable professional activities, are being implemented partly out of concerns for patient safety.^12^ If we have research that evaluates if these efforts indeed succeed in ensuring patient safety without detrimentally impacting trainees’ learning, we need to take that to our patients and the public. Are they convinced that our educational changes lead to a health care system in which they as patients would be comfortable contributing to medical education? As a field, we can enable this discussion through good research. To some, it might seem an inconvenient discussion. What if more patients opt out, choose not to be looked after by a system that educates as well as provides service? In my opinion, not having the discussion is not an option. Moreover, I am confident, supported by the limited data that are available, that the public will see the importance of being looked after by trainees, at an individual and certainly at a societal level. If everyone on the team gets the opportunity to learn from patient care, everyone, including patients, benefits.

Do our educational changes lead to a health care system in which patients comfortably contribute to medical education?

## References

[1] Cleland J , Porteous T , Skåtun D . What can discrete choice experiments do for you? Med Educ 2018;52 (11):1113–24. 30259546 10.1111/medu.13657

[2] Chaudhuri E , Mason NC , Logan S , Newbery N , Goddard AF . The Medical Registrar: Empowering the Unsung Heroes of Patient Care. London, UK: Royal College of Physicians 2013.

[3] Medical Student Involvement in Patient Care | American Medical Association [Internet]. https://www.ama-assn.org/delivering-care/medical-student-involvement-patient-care [Accessed 12 July 2018.]

[4] Ubel PA , Silver‐Isenstadt A . Are patients willing to participate in medical education? J Clin Ethics 2000;11 (3):230–5. 11127637

[5] Vaughn JL , Rickborn LR , Davis JA . Patients’ attitudes toward medical student participation across specialties: a systematic review. Teach Learn Med 2015;27 (3):245–53. 26158326 10.1080/10401334.2015.1044750

[6] Teunissen PW . Experience, trajectories, and reifications: an emerging framework of practice‐based learning in healthcare workplaces. Adv Health Sci Educ Theory Pract. 2015;20 (4):843–56. 25269765 10.1007/s10459-014-9556-y

[7] Bannister SL , Dolson MS , Lingard L , Keegan DA . Not just trust: factors influencing learners’ attempts to perform technical skills on real patients. Med Educ 2018;52 (6):605–19. 29446155 10.1111/medu.13522

[8] Berry RE , O'Dell K , Meyer BA , Purwono U . Obtaining patient permission for student participation in obstetric‐gynecologic outpatient visits: a randomized controlled trial. Am J Obstet Gynecol 2003;189 (3):634–8. 14526281 10.1067/s0002-9378(03)00876-7

[9] Burke LG , Frakt AB , Khullar D , Orav EJ , Jha AK . Association between teaching status and mortality in US Hospitals. JAMA 2017;317 (20):2105–13. 28535236 10.1001/jama.2017.5702 PMC5815039

[10] Ubel PA , Jepson C , Silver‐Isenstadt A . Don't ask, don't tell: a change in medical student attitudes after obstetrics/gynecology clerkships toward seeking consent for pelvic examinations on an anesthetized patient. Am J Obstet Gynecol 2003;188 (2):575–9. 12592274 10.1067/mob.2003.85

[11] Devettere RJ . Practical decision making in health care ethics : cases, concepts, and virtue of prudence. 657.

[12] Touchie C , Ten Cate O . The promise, perils, problems and progress of competency‐based medical education. Med Educ 2016;50 (1):93–100. 26695469 10.1111/medu.12839

